# The writer's craft

**DOI:** 10.1007/s40037-015-0176-x

**Published:** 2015-04-08

**Authors:** Lorelei Lingard

**Affiliations:** Health Sciences Addition, Schulich School of Medicine & Dentistry- Western University, Room 112, N6A 5C1 London, ON Canada

A medical education research colleague asked me for career advice recently, and in the course of our conversation I asked her, ‘How’s your writing?’ I didn’t intend it to be a loaded question, but it clearly was: she squirmed in her seat and avoided meeting my gaze. The pause stretched awkwardly as if I’d asked an intimate question like ‘How’s your marriage?’, and she was deciding how to handle this faux pas. Finally she answered, ‘I guess I’m okay at it…I don’t know…I’m not one of those people who’s a *writer*, you know? I sort of hate it.’

For researchers, writing is both intensely private and inevitably public. Most writing happens in isolation—picture yourself sitting at your laptop, alternating between typing furiously and leaning on the delete key in frustration as you shape your research into a timely, compelling, defensible story. But although it is a lonely act, the aim of research writing is public consumption—researchers write because we’re in the business of knowledge creation. We create knowledge not only through systematic research methods and eureka moments, but also through acts of argument and persuasion, negotiation and disagreement. Medical education moves forward because we share insights, question methods, argue the relevance of emerging ideas and build on one another’s efforts. All of this is possible in large part because of writing, and it explains why writing is such a highly valued currency in research.

Interestingly, many researchers feel that this currency is in short supply in their own pockets. And since effective writing is a skill many feel they lack, the question ‘How’s your writing?’ gets at one of the dark hearts of imposter syndrome among medical education researchers. And, let’s be frank, a lot of our scientific writing is *terrible*: dry, chalky, convoluted stuff that even an engaged reader struggles to choke down. We find ourselves in a troubling situation: one of our valued practices—writing—is highly fraught, both because many individual writers feel unskilled and because our community perpetuates a shared genre we love to hate for its lack of energy, clarity and story.

What to do? There is no getting around the power of writing in science: those who write well attract attention to their work and increase their influence on knowledge creation in their field, while those who write less well struggle to be heard. But writing is not a focus of most scholars’ training in our field, and few have access to skilled writing mentors who can review their work and support them in honing the writing craft. Furthermore, there exists a belief that some people just write well and others don’t, creating a sense of futility for those struggling to join the ranks of ‘good writers’. And finally, many writers believe that research writing *has to be* dry and boring or it won’t be taken seriously—that is, it won’t be published [1].

A new section of *Perspectives*, entitled *The Writer’s Craft*, directly tackles this problem in our field. This section will offer simple tips to improve your writing in one of three areas: Energy, Clarity and Persuasiveness. Each entry will focus on one key writing feature or strategy, illustrate how it commonly goes wrong, teach the grammatical underpinnings necessary to understand it and offer suggestions to wield it effectively. I’m looking forward to leading this section and drawing on my expertise in writing pedagogy, grammar and rhetoric to spotlight key features of effective academic writing for *Perspectives* readers. This issue’s inaugural edition of *The Writer’s Craft* addresses the power of the verb. In it, I point out the habits of verb use that create sluggish prose and illustrate how to avoid these habits to produce more energetic writing.

If you’re a writer who is trying to improve, this section is for you. If you supervise others’ writing but you struggle to give clear, directive feedback, you will also find useful assistance here. Suggestions for future editions of this section can be shared on Twitter; use the hashtag #how’syourwriting? I hope you find *The Writer’s Craft* accessible, instructive and entertaining.
